# Thermographic outcomes of auricular laser acupuncture and auricular seed acupressure in older adults with chronic shoulder pain: an exploratory nonrandomized, nonconcurrent comparison

**DOI:** 10.1007/s10103-026-05078-x

**Published:** 2026-10-21

**Authors:** Gerson Quadros, Emilia Angela Lo Schiavo Arisawa, Luís Eduardo Soares

**Affiliations:** Postgraduate Program in Biomedical Engineering, Universidade do Vale do Paraíba (UNIVAP), São José dos Campos, Brazil

**Keywords:** Auriculotherapy, Low-level laser therapy, Chronic pain, Shoulder pain, Older adults, Thermography

## Abstract

Purpose: Compare pain, quality of life, and cutaneous temperature changes within predefined anterior, lateral, and posterior shoulder regions of interest between auricular laser acupuncture and seed acupressure in older adults with chronic shoulder pain. Methods: This exploratory, nonrandomized, open-label study enrolled 20 adults aged 60–90 years, allocated by order of entry to consecutive, nonconcurrent cohorts of auricular laser acupuncture (n = 10) or seed acupressure (n = 10), treated twice weekly for five weeks. VAS, McGill, SF-36, and thermography were assessed before the first (A1) and fifth sessions (A2) and after the tenth (A3) and analyzed using linear mixed-effects models. Results: From A1 to A3, VAS decreased by − 2.70 points in Laser (95% CI − 4.37 to − 1.03) and − 2.50 in Seed (95% CI − 4.17 to − 0.83); the between-group difference was − 0.20 points (Laser − Seed; 95% CI − 2.54 to 2.14). Selected McGill and SF-36 outcomes changed; McGill affective A1-to-A2 change differed between groups (Laser − Seed: −2.70; 95% CI − 4.86 to − 0.55; p = 0.017). Anterior thermal range (ΔT) showed a TIME × GROUP interaction (p = 0.007); the A1-to-A3 between-group difference was − 1.941 °C (Seed − Laser; 95% CI − 3.097 to − 0.785), although Seed ΔT was higher at baseline (+ 1.496 °C; 95% CI 0.607 to 2.385). Lateral ΔT showed a TIME effect (p = 0.033); posterior ΔT showed none. Conclusion: Clinical outcomes improved over time without evidence of differential changes between interventions. Thermography identified region-specific ΔT trajectories, warranting further investigation as a complementary method.

Clinical trial registration: ClinicalTrials.gov NCT07010627.

## Introduction

The population aged 60 years and older is projected to reach 2 billion (approximately 22% of the global population) by 2050, increasing the healthcare burden of chronic disease and disability [1].

Chronic pain, defined as pain persisting for more than three months, is particularly prevalent in older adults, with survey-based studies in Europe reporting estimates ranging from 38% to 60% among individuals aged over 65 years. Its high prevalence and impact on quality of life make chronic pain management an important clinical challenge in this population [2]. Shoulder pain has been described as the third most common musculoskeletal presentation in primary care, after low back and knee pain [3]. Its prevalence is generally higher among women, and in older adults shoulder pain may negatively affect physical function and health-related quality of life [3, 4]. Although pharmacological therapy is frequently used for pain management, polypharmacy, combined with age-related physiological changes, may increase the risk of adverse events in older adults [5].

Nonpharmacological strategies include acupuncture, whose proposed pain-modulating pathways involve nociceptive, neuroendocrine, and autonomic processes [6–8]. Auricular acupuncture stimulates specific points of the auricle through several therapeutic modalities [9].

The therapeutic response to auricular stimulation is related to the rich innervation of the auricle, whose connections with the central nervous system, particularly through vagal afferent pathways, contribute to autonomic modulation and mechanisms involved in proprioceptive processing. This neuroanatomical organization allows auricular acupuncture points to be stimulated using different modalities, including mechanical, electrical, light-based, or pressure stimulation [9]. Among these approaches, laser acupuncture uses low-level laser therapy to modulate acupuncture points through photobiomodulation. The irradiation parameters used in the present protocol (660 nm; 4 J per point) are within the range of wavelengths and energies reported in clinical studies of laser auriculotherapy for pain [10]. This non-invasive approach is associated with a low risk of adverse effects and offers a particularly favorable therapeutic profile for specific populations, including older adults and individuals with needle aversion [11]. In contrast, auricular seed acupressure provides non-invasive mechanical stimulation through repeated pressure applied to seeds fixed over selected auricular points [12]. More broadly, researchers have investigated acupuncture for its analgesic effects in treating shoulder pain in both acute and chronic conditions [13].

Infrared thermography can characterize thermal distribution in painful musculoskeletal conditions [14] and may complement clinical measures by documenting regional cutaneous responses to intervention.

Although previous studies have shown benefits of auricular seed acupressure and auricular laser acupuncture in treating painful conditions [15, 16], direct comparisons between these modalities using both clinical outcomes and thermographic assessment remain scarce. Therefore, the present exploratory study aimed to compare longitudinal changes in pain intensity and multidimensional pain characteristics, quality of life, and regional thermographic parameters between auricular laser acupuncture and auricular seed acupressure in older adults with chronic shoulder pain. No prespecified inferential hierarchy was established among the clinical and thermographic outcomes. Based on the original study hypothesis, auricular laser photobiostimulation was expected to be associated with reductions in chronic pain and improvements in quality of life.

## Methods

### Study design and participants

This was a prospective, longitudinal, exploratory, nonrandomized controlled clinical study with a quantitative approach. The study was registered on ClinicalTrials.gov (NCT07010627). The registry record was initially released for Protocol Registration and Results System (PRS) review on April 23, 2025, before participant enrollment, and was publicly posted on June 6, 2025, after enrollment had begun. The registered study comprised a broader sample of 60 older adults with chronic pain involving the shoulder, knee, or lumbar region. The shoulder-pain population was defined as part of the original study design, and the present manuscript reports the 20 participants with chronic shoulder pain included in this broader study. The sample consisted of eligible participants sequentially recruited during the data collection period. Convenience sampling was used, and no a priori sample size calculation was performed. A sensitivity analysis was therefore conducted to estimate the smallest effect size detectable with the final sample.

Individuals of both sexes aged 60–90 years with self-reported chronic shoulder pain lasting at least three months were eligible and provided written informed consent before participation. Eligibility was symptom-based and did not require a specific orthopedic diagnosis; participants were included regardless of the underlying etiology or associated comorbidities. Individuals were excluded if they were concurrently undergoing similar interventions, such as acupuncture or photobiomodulation involving the auricular region, or if anatomical or dermatological conditions prevented appropriate stimulation of the auricular acupuncture points. The original protocol listed the use of analgesic or anti-inflammatory medications as an exclusion criterion; however, during the screening period, this criterion was not applied to avoid restricting participant inclusion. This decision was made before the interventions began.

**Fig. 1:**
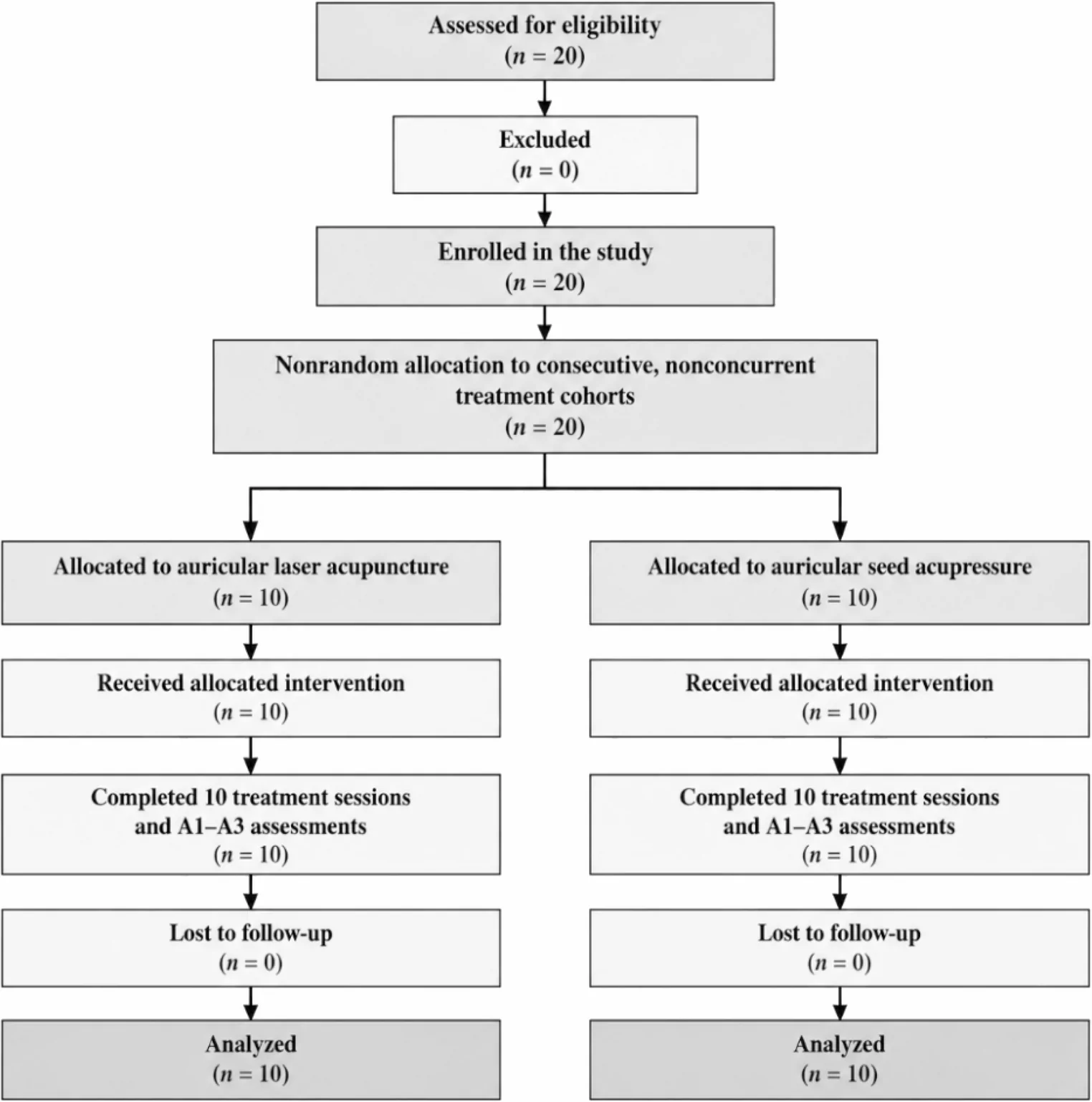
Participant flow through the study

**Fig. 2:**
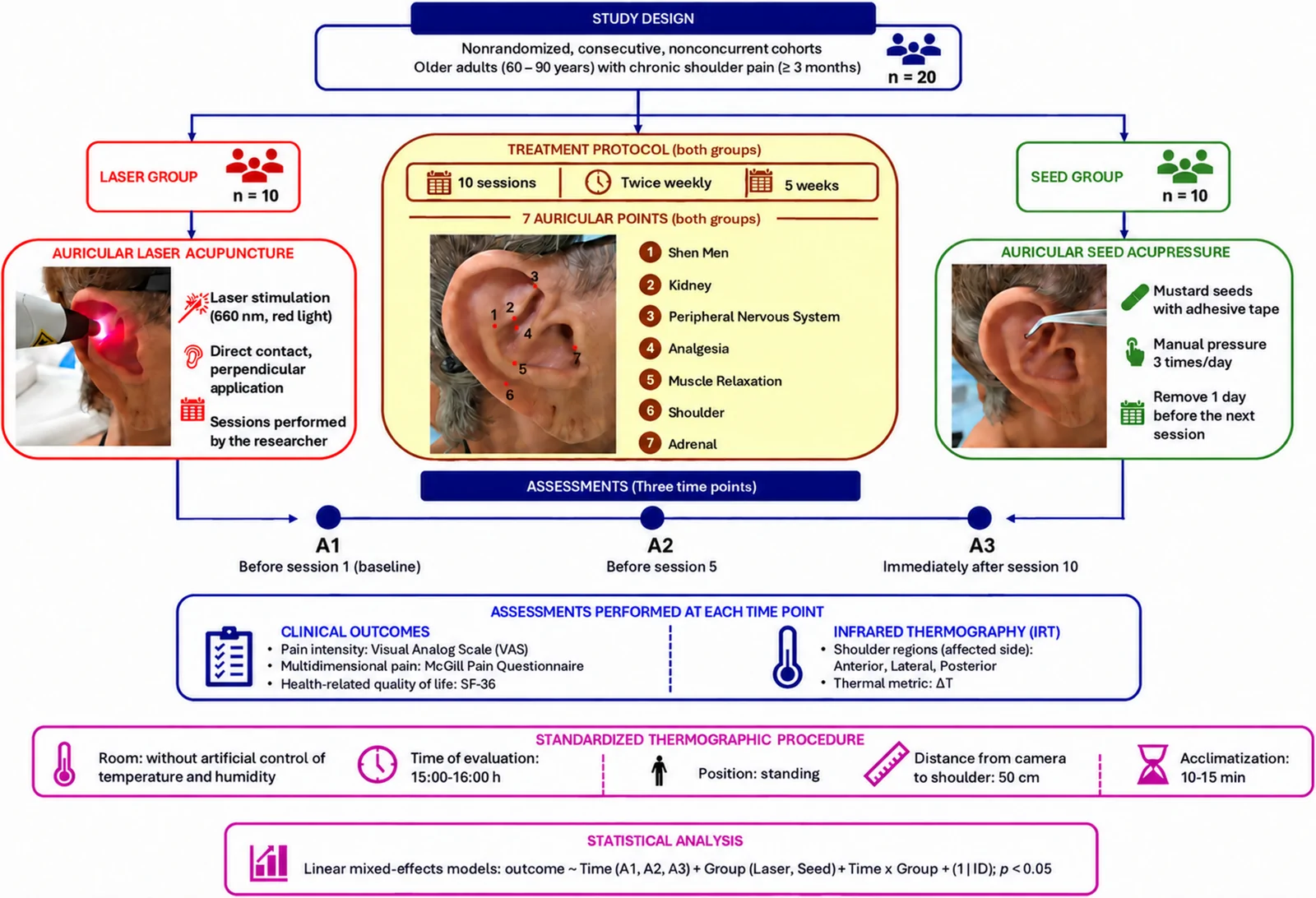
Study design, intervention protocols, auricular points, and assessment timeline

Participants were nonrandomly allocated according to order of enrollment to consecutive, nonconcurrent treatment cohorts with a predefined intervention modality. Only one treatment cohort was open for enrollment at a time, and each cohort was completed before enrollment of the subsequent cohort. Participants did not choose the intervention modality, as only the treatment assigned to the currently open cohort was available at enrollment. The cohorts were conducted sequentially as follows: Laser, May 30 to June 30, 2025; Seed, August 18 to September 19, 2025; Laser, September 29 to October 24, 2025; and Seed, October 31 to December 1, 2025. Thus, treatment allocation was determined by the intervention assigned to the cohort open at the time of enrollment rather than by randomization. The final shoulder-pain sample comprised 20 participants, 10 receiving auricular laser acupuncture and 10 receiving auricular seed acupressure. Reporting of this nonrandomized study was guided by the applicable recommendations of the TREND statement and the STRICTA guidelines for reporting acupuncture interventions [17, 18]. Participant flow through the study is presented in Fig. 1, while Fig. 2 summarizes the study design, intervention protocols, auricular points, and assessment time points.

### Intervention protocol

All participants underwent 10 intervention sessions, administered twice weekly over five consecutive weeks. Auricular stimulation was applied exclusively to the ear ipsilateral to the side with greater pain intensity, according to the predefined protocol. The principal investigator performed all interventions and had specific training and clinical experience with the technique. Before each application, the auricle was cleaned with cotton soaked in 70% ethyl alcohol.

In the Laser Group, treatment was performed using a Recover device (MM Optics^®^, São Carlos, SP, Brazil) equipped with a laser acupuncture probe. The laser was operated in continuous-wave mode at a red wavelength of 660 nm and an output power of 100 mW, according to the manufacturer’s specifications, with a beam area of 0.03 cm², irradiance of 3.33 W/cm², and radiant energy of 4 J per point (40 s of irradiation per point, corresponding to a radiant exposure of 133.3 J/cm²). Laser irradiation parameters and application procedures were reported according to recommended photobiomodulation reporting parameters [19]. The output power and beam area were based on the manufacturer’s specifications and were not independently verified using a power meter. The probe was applied in direct contact with the auricular surface and positioned perpendicular to each acupuncture point, without additional pressure. The laser was applied pointwise to the following auricular acupuncture points: Shen Men, Kidney, Peripheral Nervous System, Analgesia, Muscle Relaxation, Shoulder, and Adrenal according to the anatomical locations and therapeutic indications described by Artioli et al. [20]. During all applications, both the investigator and participants wore protective eyewear appropriate for the wavelength used, in accordance with photobiomodulation safety standards.

In the Seed Group, mustard seeds were affixed with adhesive tape to the same auricular acupuncture points. Participants were instructed to press the seeds manually three times daily and to remove them one day before the next session. New seeds and adhesive tape were applied at each treatment session. The duration and intensity of manual pressure were not standardized. Adherence to the home stimulation instructions was assessed by participant self-report during treatment sessions.

At each treatment session, participants were asked whether they had experienced any adverse reactions or problems since the previous session.

### Clinical and thermographic assessment

Clinical and thermographic assessments were performed individually in a private room at three time points: before the first session (baseline, A1), before the fifth session (A2), and immediately after the tenth session (A3). Pain intensity was assessed using the Visual Analog Scale (VAS), while its sensory and affective dimensions were evaluated using the McGill Pain Questionnaire (MPQ) proposed by Pimenta and Teixeira [21]. Health-related quality of life was assessed using the validated Brazilian Portuguese version of the 36-Item Short Form Health Survey (SF-36) [22], allowing evaluation of pain’s impact on physical and psychosocial domains. The same investigator who administered the interventions also conducted the clinical assessments, acquired the thermographic images, and subsequently delineated the ROIs and extracted the thermal data and was therefore not blinded to treatment group or assessment time point. In addition to clinical outcomes, anterior, lateral, and posterior views of the affected shoulder were assessed using a FLIR ONE Pro LT Gen 3 infrared thermal camera (Teledyne FLIR LLC, Wilsonville, OR, USA) connected to a mobile device, with skin emissivity set to 0.98 for all acquisitions. The camera performed automatic internal non-uniformity correction (NUC) during operation; no external calibration procedure was performed. Standardization of acquisition conditions and image analysis is recommended to improve the consistency of human skin temperature measurements obtained by infrared thermography [23].

Thermal images were acquired in an examination room between 3:00 and 4:00 p.m. under ambient temperature and humidity conditions, without artificial control of these environmental variables. Room temperature, relative humidity, and airflow were not systematically recorded. Before image acquisition, participants remained in the examination room for a 10–15 min acclimatization period. Participants were instructed to avoid physical exercise, bathing, alcohol consumption, and stimulants such as caffeine for two hours before the examination. During image acquisition, participants remained standing upright on a rubberized surface, with the region of interest free of clothing and accessories. The distance between the thermal camera and the region of interest was standardized at 50 cm to ensure consistent framing across assessments.

### Thermographic image acquisition and processing

The original radiometric FLIR files were transferred from the camera to FLIR Ignite and subsequently stored on a computer for analysis. The radiometric files were processed using Fiji/ImageJ (National Institutes of Health, USA) with ThermImageJ. Radiometric temperature data were converted into 32-bit temperature images in which pixel values represented temperature in degrees Celsius (°C), rather than grayscale intensity values, thereby preserving pixel-level temperature information for quantitative analysis. The regions of interest (ROIs) were anatomically standardized for the anterior, lateral, and posterior shoulder views. The anterior ROI covered the anterior deltoid between the lateral clavicle and the level of the anterior axillary fold; the lateral ROI encompassed the middle deltoid between the acromion and the level of the axillary fold; and the posterior ROI covered the posterior deltoid between the scapular spine and the level of the posterior axillary fold. For each ROI, maximum temperature (Tmax) and minimum temperature (Tmin) were extracted, and the regional thermal range was calculated as ΔT = Tmax − Tmin for statistical analysis.

### Data analysis

Data obtained from the clinical and thermographic assessments were analyzed using Jamovi (version 2.6.44.0) with the GAMLj Mixed Models module. All 20 participants completed the three assessment time points and were included in the analyses, with no missing outcome data. Dependent outcomes were analyzed using linear mixed-effects models (LMMs), including TIME (A1, A2, and A3), GROUP (Laser and Seed), and the TIME × GROUP interaction as fixed effects. Participant was included as a random effect (random intercept) to account for within-participant dependence arising from repeated measurements. Parameter estimation was performed using restricted maximum likelihood (REML) with the BOBYQA optimization algorithm. Degrees of freedom were calculated using the Satterthwaite approximation, and the significance of fixed effects was assessed using F-tests.

Model adequacy was assessed through graphical inspection and normality tests of the residuals. Significant omnibus effects were further explored using comparisons of estimated marginal means (EMMs) and, when appropriate, simple-effects analyses, with Bonferroni adjustment for multiple comparisons. Statistical significance was set at 5% (*p* < 0.05). Model estimates and relevant contrasts were reported with their corresponding 95% Wald confidence intervals. No single primary outcome was prospectively designated for the present shoulder analysis; therefore, the analyses were considered exploratory, and no global multiplicity adjustment across outcomes was applied.

A post hoc sensitivity analysis was performed for the available sample (*n* = 20; 10 participants per group; three time points), assuming α = 0.05 and 80% power and using variance components estimated from the linear mixed-effects models for the VAS and ΔT (Tmax − Tmin) in the anterior, lateral, and posterior shoulder regions. For the TIME × GROUP interaction, a progressive linear divergence between trajectories was assumed, corresponding to 50% of the final between-group difference at A2 and 100% at A3 relative to A1. Under these assumptions, the analysis indicated detectable final between-group differences of approximately 3.8 points on the VAS and 1.05–1.87 °C in ΔT, depending on the shoulder region assessed.

## Results

All 20 participants (10 per group) completed treatment and assessments without missing data, loss to follow-up, or adverse-event-related interruptions or withdrawals. No adverse events were reported. Mean age was 71.0 ± 6.1 years (range 61–83); 18 participants (90%) were women. Baseline characteristics are shown in Table 1.

**Table 1:** Baseline demographic and clinical characteristics of participants according to treatment group

Characteristic	Laser (*n* = 10)	Seed (*n* = 10)	Total (*n* = 20)
Age, years, mean ± SD	73.5 ± 6.0	68.4 ± 5.3	71.0 ± 6.1
Female sex, n (%)	10 (100%)	8 (80%)	18 (90%)
Male sex, n (%)	0 (0%)	2 (20%)	2 (10%)
Right painful shoulder, n (%)	7 (70%)	6 (60%)	13 (65%)
Left painful shoulder, n (%)	3 (30%)	4 (40%)	7 (35%)
Baseline VAS, mean ± SD	7.9 ± 1.73	7.3 ± 1.89	7.6 ± 1.79

Note: Data are presented as mean ± standard deviation (SD) or n (%), as appropriate. *VAS*, visual analog scale

**Fig. 3:**
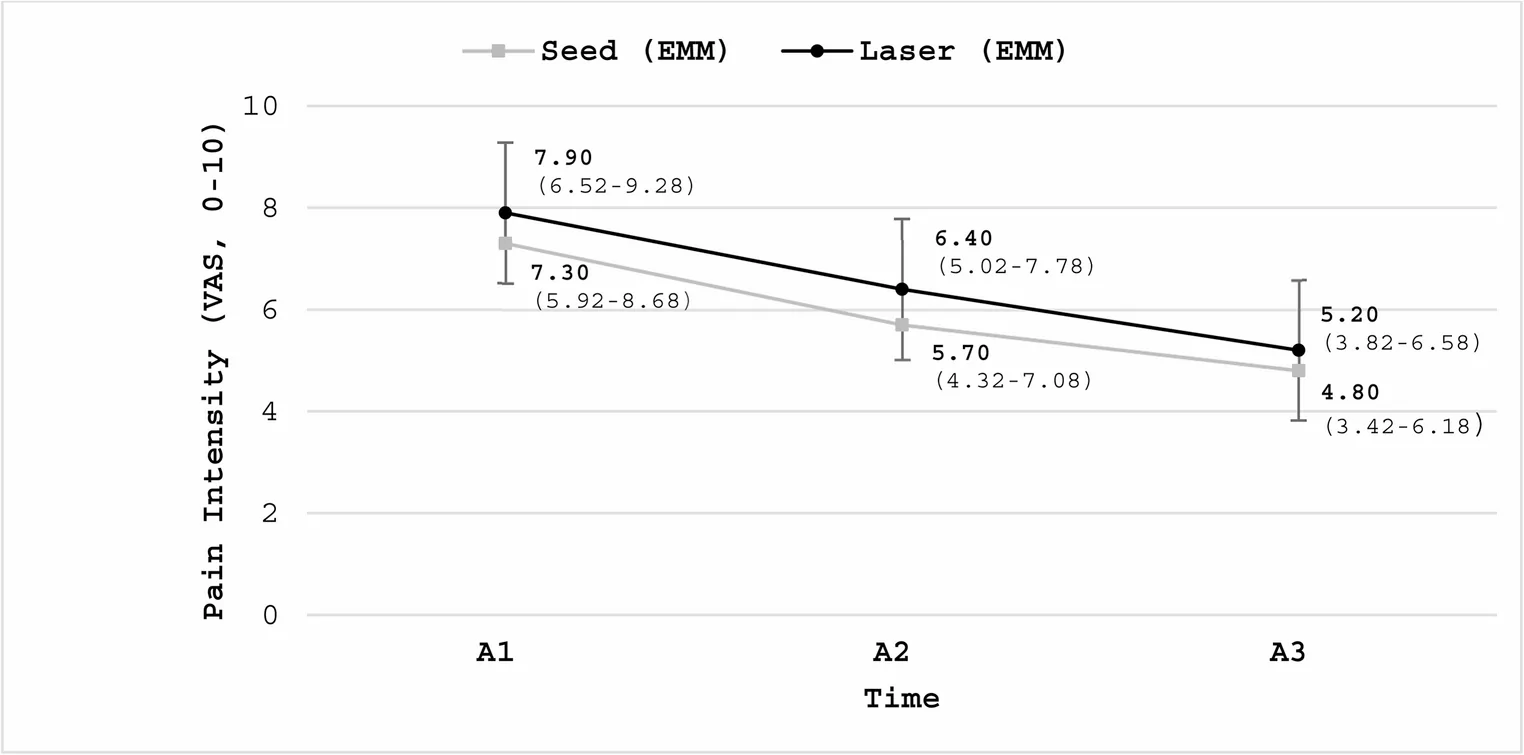
Estimated marginal means of VAS by treatment group and time point. Note. Points represent the estimated marginal means (EMMs) obtained from the linear mixed-effects model, and error bars represent 95% confidence intervals (95% CIs). Lower VAS scores indicate lower pain intensity. The adjusted between-group contrast in A3 − A1 change (Laser − Seed) was − 0.20 VAS points (95% CI − 2.54 to 2.14; *p* = 0.865)

VAS declined over time (TIME: F = 10.04; p < 0.001), without GROUP (F = 0.66; p = 0.428) or TIME × GROUP effects (F = 0.03; p = 0.966; Table 2). Bonferroni-adjusted comparisons showed reductions from A1 to A2 (p = 0.035) and A1 to A3 (p < 0.001), but not A2 to A3 (p = 0.241). Table 3 reports group-specific estimated marginal means, A1-to-A3 changes, and the between-group contrast with 95% CIs (Fig. 3)

**Table 2:** Omnibus tests for VAS pain scores

Effect	F	df	*P*
Time	10.04	2; 36	< 0.001*
Group	0.66	1; 18	0.428
Time × Group	0.03	2; 36	0.966

Note. Values refer to the omnibus tests of the linear mixed-effects model, with TIME, GROUP, and the TIME × GROUP interaction included as fixed effects and participant as a random intercept. Degrees of freedom were estimated using the Satterthwaite approximation. Post-hoc comparisons between time points were adjusted using the Bonferroni method. Statistically significant values are shown in bold and indicated by an asterisk (*)

**Table 3:** Estimated VAS scores and changes by treatment group

Group	A1	A2	A3	A3 − A1 change
Laser (*n* = 10)	7.90 (6.52–9.28)	6.40 (5.02–7.78)	5.20 (3.82–6.58)	−2.70 (− 4.37 to − 1.03)
Seed (*n* = 10)	7.30 (5.92–8.68)	5.70 (4.32–7.08)	4.80 (3.42–6.18)	−2.50 (− 4.17 to − 0.83)

Note. Values at A1, A2, and A3 are estimated marginal means (EMMs), with 95% Wald confidence intervals (CIs) shown in parentheses. A3 − A1 represents the model-estimated change from baseline to the final assessment, with the corresponding 95% CI. The adjusted between-group contrast in A3 − A1 change (Laser − Seed) was − 0.20 VAS points (95% CI − 2.54 to 2.14; *p* = 0.865)

Data from the McGill Pain Questionnaire showed different response patterns across assessment time points (Table 4). The sensory IAvD and total PRI(T) scores showed significant effects of TIME (*p* = 0.004 and *p* = 0.010, respectively), with no significant effects of GROUP or TIME × GROUP interaction. For IAvD, the estimated marginal mean decreased from 22.35 at A1 to 17.90 at A3, with significant differences between A1 and A3 (*p* = 0.008) and between A2 and A3 (*p* = 0.019). For PRI(T), the estimated marginal mean decreased from 40.20 at A1 to 33.00 at A3, with a significant difference between A1 and A3 (*p* = 0.010).

Among the specific domains, the affective component (AF) showed a significant GROUP effect (*p* = 0.035) and a significant TIME × GROUP interaction (*p* = 0.041). The adjusted between-group contrast in change was significant from A1 to A2 (Laser − Seed: −2.70; 95% CI − 4.86 to − 0.55; *p* = 0.017), but not from A1 to A3 (− 2.10; 95% CI − 4.26 to 0.06; *p* = 0.058) (Table 5). M(AfAv) showed no significant effects. M(T) showed a TIME effect (*p* = 0.046), without significant GROUP or interaction effects; no pairwise TIME comparison was significant after Bonferroni adjustment (A1–A2: *p* = 1.000; A1–A3: *p* = 0.056; A2–A3: *p* = 0.190).

**Table 4:** Omnibus tests of the linear mixed-effects models for McGill Pain Questionnaire scores

Outcome	Time (*p*)	Group (*p*)	Interaction (*p*)
IAvD	0.004*	0.270	0.193
PRI(T)	0.010*	0.377	0.282
AF	0.288	0.035*	0.041*
M(AfAv)	0.088	0.632	0.535
M(T)	0.046*	0.293	0.682

Note. Values represent p-values from the omnibus tests of the linear mixed-effects models for the fixed effects of TIME, GROUP, and the TIME × GROUP interaction. Statistically significant values are shown in bold and indicated by an asterisk (*). Scores sum the selected descriptor ranks: *IAvD* sensory groups 1–10, *AF* affective groups 11–15, *M(AfAv)* group 20, *M(T)* miscellaneous groups 17–20, *PRI(T)* total groups 1–20

**Table 5:** Estimated McGill Pain Questionnaire scores and model-based changes according to treatment group

Scale	Group	A1	A2	A3	A3 − A1 change
IAvD	Seed	20.0 (16.4–23.6)	20.7 (17.1–24.3)	18.1 (14.5–21.7)	−1.90 (− 5.85 to 2.05)
	Laser	24.7 (21.1–28.3)	23.1 (19.5–26.7)	17.7 (14.1–21.3)	−7.00 (− 10.95 to − 3.05)
PRI(T)	Seed	36.7 (31.0–42.4)	37.8 (32.1–43.5)	32.8 (27.1–38.5)	−3.90 (− 10.50 to 2.70)
	Laser	43.7 (38.0–49.4)	38.5 (32.8–44.2)	33.2 (27.5–38.9)	−10.50 (− 17.10 to − 3.90)
AF	Seed	6.60 (5.45–7.75)	7.40 (6.25–8.55)	6.80 (5.65–7.95)	+ 0.20 (− 1.34 to 1.74)
	Laser	7.00 (5.85–8.15)	5.10 (3.95–6.25)	5.10 (3.95–6.25)	−1.90 (− 3.44 to − 0.36)

Note. Each treatment group included 10 participants. Values at A1, A2, and A3 are estimated marginal means (EMMs), with 95% Wald confidence intervals (CIs) shown in parentheses. A3 − A1 represents the model-estimated change from baseline to A3, with the corresponding 95% CI. The adjusted between-group contrasts in A3 − A1 change (Laser − Seed) were − 5.10 for IAvD (95% CI − 10.62 to 0.42; *p* = 0.072), − 6.60 for PRI(T) (95% CI − 15.84 to 2.64; *p* = 0.160), and − 2.10 for AF (95% CI − 4.26 to 0.06; *p* = 0.058). For AF, the adjusted between-group contrast in A2 − A1 change was − 2.70 (95% CI − 4.86 to − 0.55; *p* = 0.017)

In the 36-Item Short Form Health Survey (SF-36), the domains showed different patterns across assessment time points (Table 6). Significant effects of TIME were observed for Physical Functioning (PF), Role Limitations due to Physical Health (RP), Bodily Pain (BP), General Health (GH), Vitality (VT), and Role Limitations due to Emotional Problems (RE). Bonferroni-adjusted multiple comparisons showed significant differences between A1 and A3 for PF (*p* = 0.006), RP (*p* = 0.031), BP (*p* = 0.002), GH (*p* = 0.009), VT (*p* = 0.004), and RE (*p* = 0.005). In addition, BP and VT differed significantly between A1 and A2 (*p* = 0.020 and *p* = 0.042, respectively).

Only the BP domain showed a significant GROUP effect (*p* = 0.022). No significant omnibus TIME × GROUP interactions were observed for any SF-36 domain (Table 6). Group-specific estimated marginal means, changes from A1 to A3, and between-group contrasts in change, with their corresponding 95% confidence intervals, are presented in Table 7. For BP, the between-group contrast in A1-to-A3 change was + 18.10 points (95% CI 0.51 to 35.69; *p* = 0.046), despite the non-significant omnibus TIME × GROUP interaction. The Social Functioning (SF) and Mental Health (MH) domains showed no significant effects of TIME, GROUP, or the TIME × GROUP interaction.

**Table 6:** Omnibus tests of the linear mixed-effects models for the SF-36 domains

Outcome		Time (*p*)	Group (*p*)	Interaction (*p*)
PF	0.007*		0.294	0.570
RP	0.033*		0.160	0.904
BP	0.002*		0.022*	0.094
GH	0.029*		0.210	0.956
VT	0.013*		0.303	0.162
SF	0.303		0.439	0.990
RE	0.017*		0.528	0.648
MH	0.083		0.972	0.370

Note. Values represent *p*-values from the omnibus tests of the linear mixed-effects models for the fixed effects of TIME, GROUP, and the TIME × GROUP interaction. Degrees of freedom were estimated using the Satterthwaite approximation. Statistically significant values are shown in bold and indicated by an asterisk (*). *PF* = Physical Functioning, *RP* = Role Limitations due to Physical Health, *BP* = Bodily Pain, *GH* = General Health, *VT* = Vitality, *SF* = Social Functioning, *RE* = Role Limitations due to Emotional Problems, *MH* = Mental Health

**Table 7:** Estimated SF-36 domain scores and model-based changes according to treatment group

Domain	Group	A1	A2	A3	A3 − A1 change
PF	Seed	57.0 (42.5–71.5)	60.0 (45.5–74.5)	66.5 (52.0–81.0)	+ 9.50 (− 2.57 to 21.60)
	Laser	43.0 (28.5–57.5)	50.5 (36.0–65.0)	61.5 (47.0–76.0)	+ 18.50 (6.43 to 30.60)
RP	Seed	42.5 (15.9–69.1)	57.5 (30.9–84.1)	70.0 (43.4–96.6)	+ 27.50 (− 5.63 to 60.60)
	Laser	17.5 (− 9.1–44.1)	42.5 (15.9–69.1)	52.5 (25.9–79.1)	+ 35.00 (1.87 to 68.10)
BP	Seed	49.4 (38.8–60.0)	54.1 (43.5–64.7)	56.6 (46.0–67.2)	+ 7.20 (− 5.37 to 19.80)
	Laser	24.3 (13.7–34.9)	44.8 (34.2–55.4)	49.6 (39.0–60.2)	+ 25.30 (12.73 to 37.90)
GH	Seed	46.6 (33.3–59.9)	50.7 (37.4–64.0)	56.4 (43.1–69.7)	+ 9.80 (− 1.51 to 21.10)
	Laser	56.0 (42.7–69.3)	60.8 (47.5–74.1)	68.1 (54.8–81.4)	+ 12.10 (0.79 to 23.40)
VT	Seed	52.5 (42.3–62.7)	61.0 (50.8–71.2)	58.0 (47.8–68.2)	+ 5.50 (− 5.72 to 16.70)
	Laser	42.5 (32.3–52.7)	50.5 (40.3–60.7)	61.0 (50.8–71.2)	+ 18.50 (7.28 to 29.70)
RE	Seed	36.7 (9.4–63.9)	60.0 (32.8–87.2)	63.3 (36.1–90.6)	+ 26.70 (− 0.32 to 53.70)
	Laser	30.0 (2.8–57.2)	40.0 (12.8–67.2)	60.0 (32.8–87.2)	+ 30.00 (3.01 to 57.00)

Note. Each group included 10 participants. Values are estimated marginal means (EMMs) with 95% Wald confidence intervals (CIs) in parentheses. A3 − A1 represents the model-estimated change from A1 to A3. Between-group contrasts in A3 − A1 change (Laser − Seed) were: PF, + 9.00 (− 7.90 to 25.90; *p* = 0.292); RP, + 7.50 (− 38.86 to 53.86; *p* = 0.747); BP, + 18.10 (0.51 to 35.69; *p* = 0.046); GH, + 2.30 (− 13.53 to 18.10; *p* = 0.772); VT, + 13.00 (− 2.70 to 28.70; *p* = 0.105); and RE, + 3.33 (− 34.43 to 41.10; *p* = 0.861)

**Fig. 4:**
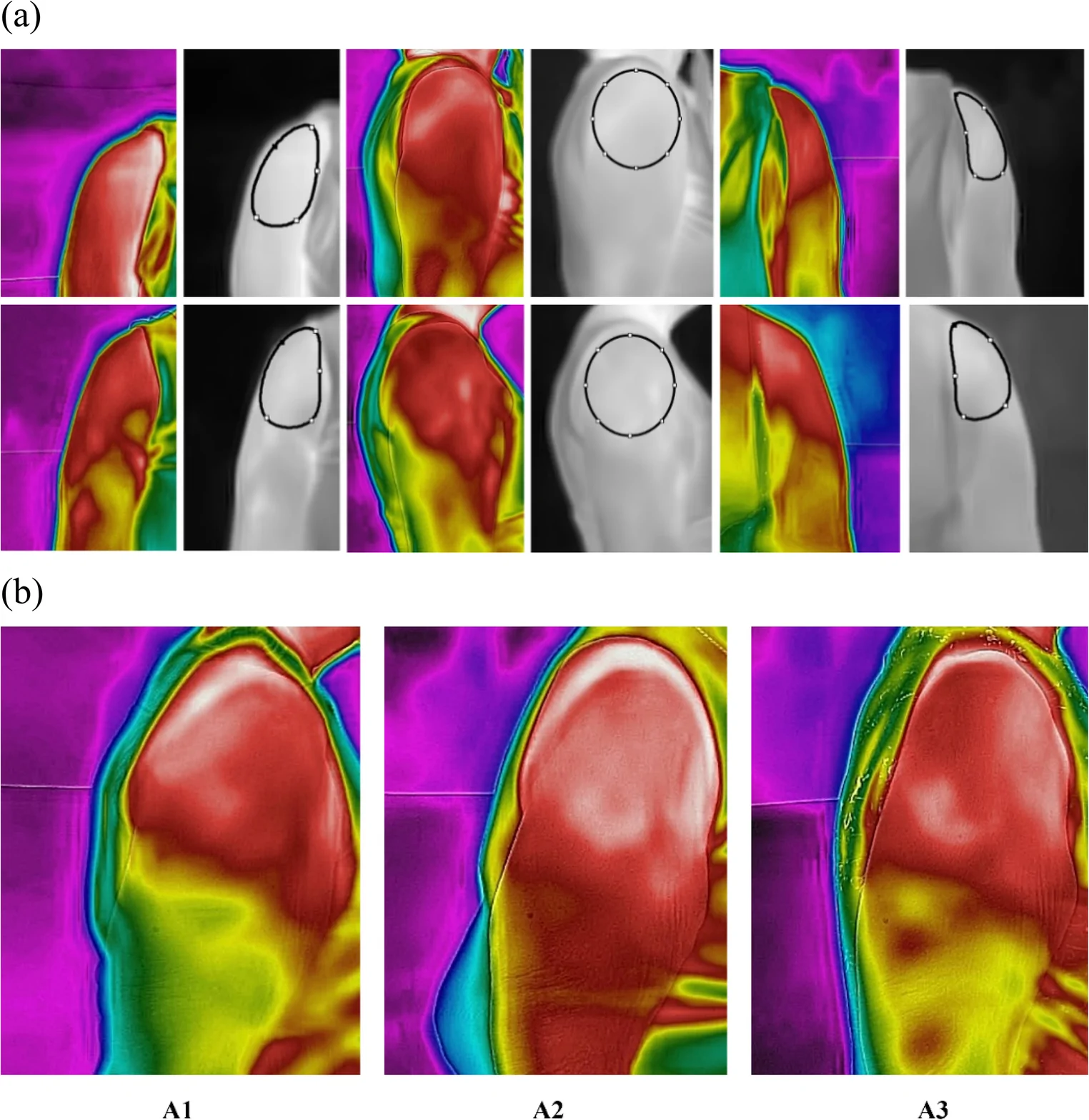
(**a**) Example of the delineation of regions of interest (ROIs) in the anterior, lateral, and posterior shoulder regions for thermographic analysis. Note. Pseudocolor thermographic images are shown alongside the corresponding 32-bit temperature images processed in Fiji/ImageJ using ThermImageJ, in which pixel values represent temperature in degrees Celsius (°C). The outlined areas represent the regions of interest (ROIs) used for quantitative thermal analysis. (**b**) Pseudocolor thermographic images of the lateral shoulder region from the same participant at different assessment time points. Note. A1: before the first session; A2: before the fifth session; A3: immediately after the tenth session. Pseudocolor thermographic images are presented for illustrative purposes only and should not be used for visual comparison of temperature across assessment time points. Quantitative thermal analyses were performed using the corresponding 32-bit temperature images processed in Fiji/ImageJ with ThermImageJ

Regional shoulder ΔT was analyzed anteriorly, laterally, and posteriorly (Fig. 4a and b). 

Thermographic findings differed across the shoulder regions (Tables 8 and 9). In the anterior region, a significant TIME × GROUP interaction was observed (*p* = 0.007), whereas the main effects of TIME (*p* = 0.327) and GROUP (*p* = 0.096) were not significant. The Seed group showed significant reductions in ΔT from A1 to A2 (− 0.940 °C, 95% CI − 1.766 to − 0.114; *p* = 0.027) and from A1 to A3 (− 1.223 °C, 95% CI − 2.049 to − 0.397; *p* = 0.005), whereas the corresponding changes in the Laser group were not significant. However, a significant baseline difference was present, with higher ΔT in the Seed group than in the Laser group (difference, + 1.496 °C; 95% CI 0.607 to 2.385; *p* = 0.001).

In the lateral region, a significant effect of TIME was observed (*p* = 0.033), whereas the GROUP effect (*p* = 0.770) and the omnibus TIME × GROUP interaction (*p* = 0.067) were not significant. From A1 to A3, ΔT decreased in the Seed group (− 0.676 °C, 95% CI − 1.141 to − 0.211; *p* = 0.006), whereas no change was observed in the Laser group (+ 0.020 °C, 95% CI − 0.446 to 0.485; *p* = 0.932). The between-group contrast in A1-to-A3 change was + 0.696 °C (Laser − Seed; 95% CI 0.045 to 1.347; *p* = 0.039), despite the non-significant omnibus interaction. No significant between-group difference was present at baseline (*p* = 0.467).

In the posterior region, no significant effects of TIME (*p* = 0.302), GROUP (*p* = 0.429), or TIME × GROUP interaction (*p* = 0.115) were observed.

**Table 8:** Omnibus tests of the linear mixed-effects models for regional thermal range (ΔT)

Region	Time (*p*)	Group (*p*)	Interaction (*p*)
Anterior	0.327	0.096	0.007*
Lateral	0.033*	0.770	0.067
Posterior	0.302	0.429	0.115

Note. Values represent *p*-values from the omnibus tests of the linear mixed-effects models for the fixed effects of TIME, GROUP, and the TIME × GROUP interaction. Degrees of freedom were estimated using the Satterthwaite approximation. Statistically significant values are shown in bold and indicated by an asterisk (*). ΔT = regional thermal range (Tmax − Tmin)

**Table 9:** Estimated regional thermal range (ΔT) and model-based changes according to treatment group

Region	Group	A1, °C	A2, °C	A3, °C	A3 − A1, °C
Anterior	Seed	4.27 (3.64–4.90)	3.33 (2.70–3.96)	3.05 (2.42–3.68)	−1.223 (− 2.049 to − 0.397)
	Laser	2.78 (2.15–3.40)	2.85 (2.22–3.47)	3.49 (2.87–4.12)	+ 0.718 (− 0.108 to 1.544)
Lateral	Seed	2.15 (1.78–2.52)	1.71 (1.34–2.08)	1.47 (1.10–1.84)	−0.676 (− 1.141 to − 0.211)
	Laser	1.96 (1.59–2.33)	1.55 (1.18–1.92)	1.98 (1.61–2.35)	+ 0.020 (− 0.446 to 0.485)
Posterior	Seed	2.40 (1.86–2.94)	2.32 (1.78–2.86)	2.17 (1.63–2.71)	−0.228 (− 0.802 to 0.347)
	Laser	2.62 (2.08–3.16)	2.15 (1.61–2.69)	2.85 (2.31–3.39)	+ 0.236 (− 0.339 to 0.810)

Note. Each group included 10 participants. Values at A1, A2, and A3 are estimated marginal means (EMMs) with 95% Wald confidence intervals (CIs) in parentheses. A3 − A1 represents the model-estimated change from baseline to the final assessment. For the anterior region, the baseline between-group contrast (Seed − Laser) was + 1.496 °C (95% CI 0.607 to 2.385; *p* = 0.001), and the between-group contrast in A1-to-A3 change (Seed − Laser) was − 1.941 °C (95% CI − 3.097 to − 0.785; *p* = 0.002). For the lateral region, the baseline contrast (Laser − Seed) was − 0.190 °C (95% CI − 0.712 to 0.332; *p* = 0.467), and the between-group contrast in A1-to-A3 change (Laser − Seed) was + 0.696 °C (95% CI 0.045 to 1.347; *p* = 0.039). No significant between-group contrasts were observed in the posterior region

## Discussion

In this comparison of auricular laser photobiomodulation (PBM) and seed acupressure in older adults with chronic shoulder pain, clinical outcomes improved over time without consistent between-group differences. Thermographic responses were region-dependent: anterior ΔT showed a TIME × GROUP interaction with baseline imbalance, lateral ΔT a TIME effect without an omnibus interaction, and posterior ΔT no significant effects. These superficial thermal observations do not establish distinct physiological mechanisms.

From a translational perspective, clinical outcomes improved over the treatment period. Pain intensity (VAS) decreased significantly from baseline (A1) to mid-treatment (A2) and remained reduced at the final assessment (A3), whereas McGill PRI improved significantly from baseline (A1) to A3. Although MCID values have been reported for pain measures in shoulder disorders, these thresholds vary according to the underlying diagnosis and study population and may not be directly applicable to heterogeneous populations with shoulder pain [24]. No consistent differences in clinical response were observed between the intervention groups. The auricle is densely innervated by branches of the vagus, trigeminal, and cervical spinal nerves [9], providing a neuroanatomical basis for the potential modulation of nociceptive signaling by auricular stimulation. Central gating and autonomic mechanisms have been proposed as possible pathways through which auricular stimulation may influence pain processing; however, these mechanisms were not directly assessed in the present study. The thermographic measurements therefore provide complementary information on superficial thermal patterns rather than direct evidence of the neurophysiological mechanisms underlying the observed clinical changes.

Infrared thermography measures superficial skin temperature, and ΔT = Tmax − Tmin characterizes the range within an ROI [25]. Here, ΔT patterns varied by shoulder region. The anterior TIME × GROUP interaction warrants caution because Seed ΔT was significantly higher than Laser ΔT at baseline. Although Seed ΔT declined from A1 to A3, this imbalance limits attribution of the later trajectories to treatment. Lateral ΔT showed a TIME effect and a Seed-group reduction from A1 to A3, but its omnibus TIME × GROUP interaction was not significant. The posterior region showed no significant effects. Thus, the results describe heterogeneous superficial thermal distribution without establishing vascular, inflammatory, or autonomic mechanisms.

Photonic laser energy and mechanical seed pressure differ, but these thermal data cannot determine whether stimulus type caused the regional patterns. Proposed pathways provide hypotheses for future mechanistic studies.

For the Laser intervention, PBM at 660 nm represents a photonic mode of stimulation. Proposed photobiological mechanisms of PBM include photon absorption by mitochondrial chromophores, particularly cytochrome c oxidase, followed by modulation of cellular signaling pathways involving ATP production, reactive oxygen species, and nitric oxide [26]. These mechanisms have been discussed in the context of laser acupuncture and pain modulation [26], but they were not directly measured in the present study. Accordingly, the observed thermographic patterns cannot be attributed specifically to mitochondrial, microvascular, or nitric oxide-mediated responses.

For the Seed intervention, repeated manual pressure represents a mechanical mode of auricular stimulation. Auricular point acupressure using seeds provides sustained stimulation of auricular points, and recent randomized clinical evidence in older adults supports repeated self-administered pressure as a component of this therapeutic approach [12]. However, the specific neural and autonomic pathways involved were not directly assessed in the present study. Moreover, because the reduction in anterior ΔT in the Seed group occurred in the presence of a significant baseline between-group difference, this thermal change should not be interpreted as evidence of a specific mechanically mediated physiological response.

Standardized ROI processing in Fiji/ImageJ allows noncontact quantification of superficial thermal patterns. Emissivity (0.98), acquisition distance (50 cm), timing, posture, and acclimatization were standardized, but IRT remains sensitive to environmental, physiological, and acquisition factors. Regional ΔT may complement patient-reported outcomes; it does not establish a neuromodulation biomarker or distinguish mechanisms between interventions.

Methodological limitations should be considered. The small sample size (*n* = 20) limited the precision of between-group comparisons, although the sensitivity analysis indicated detectable changes of approximately 3.8 points for VAS and 1.05–1.87 °C for ΔT under the specified model assumptions. Participants were allocated according to order of enrollment into sequential, non-concurrent cohorts rather than by randomization, resulting in predictable allocation based on enrollment order and potential temporal and environmental confounding, as room temperature and humidity were not artificially controlled. This is particularly relevant to the baseline imbalance observed in anterior ΔT. Sequential recruitment may also have introduced cohort-period differences, and potential investigator-related procedural variation over time cannot be fully excluded. The open-label design and absence of a sham or inactive control may have contributed to contextual or expectancy effects on patient-reported outcomes. The lack of investigator blinding during clinical and thermographic assessments, including ROI delineation, may have introduced performance and detection bias. In addition, participants were enrolled based on chronic shoulder pain rather than a specific musculoskeletal diagnosis, and the two modalities inherently differed in their patterns of stimulus exposure; these factors limit the attribution of between-group differences to the interventions themselves and should be considered when generalizing the findings.

IRT is limited to the two-dimensional measurement of superficial cutaneous temperature; consequently, the physiological mechanisms discussed remain hypothetical. Multiple clinical and thermographic outcomes were analyzed, with Bonferroni adjustment applied to post-hoc comparisons but without global adjustment across outcomes, increasing the possibility of type I error and supporting an exploratory interpretation of the findings. Finally, because A3 was obtained at the end of the treatment period, after the tenth session, the persistence of the observed clinical and thermal changes beyond the intervention period cannot be established.

Future studies should use larger randomized samples, controlled or continuously monitored environmental conditions, and follow-up assessments beyond the treatment period. The integration of IRT with complementary physiological measurements, such as laser Doppler flowmetry, heart rate variability (HRV), multispectral optical imaging, or dynamic thermal stress testing, may help determine whether regional thermal changes are associated with alterations in cutaneous perfusion or autonomic regulation. Such multimodal approaches could also clarify whether photonic and mechanical auricular stimulation produce distinct physiological responses and how these responses relate to clinical outcomes.

## Conclusion

In this exploratory nonrandomized, non-concurrent comparison, the observed reductions in pain and improvements in quality-of-life outcomes over the treatment period were consistent with the original hypothesis that auricular laser photobiostimulation would be associated with clinical improvement in older adults with chronic shoulder pain. However, no consistent evidence of differential clinical responses between auricular laser acupuncture and auricular seed acupressure was observed, and the study design does not permit conclusions regarding treatment equivalence or intervention-specific efficacy.

Infrared thermography identified region-dependent changes in superficial cutaneous thermal patterns, particularly in anterior and lateral ΔT; however, the anterior finding occurred in the presence of a significant baseline between-group difference. These thermographic observations should therefore be considered exploratory and do not establish intervention-specific physiological mechanisms. Within these limitations, IRT showed feasibility as a complementary, non-contact method for characterizing superficial thermal variation alongside clinical outcomes.

## Data Availability

The datasets generated and/or analyzed during the current study are not publicly available due to participant privacy and confidentiality considerations but are available from the corresponding author on reasonable request.
